# Vitrification has detrimental effects on maturation, viability, and subcellular quality of oocytes post IVM in cancerous women: An experimental study

**DOI:** 10.18502/ijrm.v17i3.4516

**Published:** 2019-05-29

**Authors:** Mehdi Mohsenzadeh, Nasim Tabibnejad, Mahboubeh Vatanparast, Fatemeh Anbari, Mohammad Ali Khalili, Mojgan Karimi-Zarchi

**Affiliations:** ^1^Research and Clinical Center for Infertility, Shahid Sadoughi University of Medical Sciences, Yazd, Iran.; ^2^Gerash Al-Zahra Fertility Center, Gerash University of Medical Sciences, Gerash, Iran.; ^3^Rafsanjan University of Medical Sciences, Rafsanjan, Iran.; ^4^Department of Obstetrics and Gynecology, Shahid Sadoughi Hospital, Faculty of Medicine, Shahid Sadoughi University of Medical Sciences, Yazd, Iran.; ^5^Iran University of Medical Sciences, Tehran, Iran.

**Keywords:** *IVM*, * Vitrification*, * Fertility preservation*, * Maturation rate*, * Meiotic spindle*, * Zona pellucida birefringence.*

## Abstract

**Background:**

In vitro maturation (IVM) of immature oocytes retrieved from ovarian tissue has been considered as a valuable approach for fertility preservation in cancerous patients.

**Objective:**

To evaluate the efficacy of vitrification on oocyte maturation, survival rates, as well as the subcellular oocyte quality post IVM.

**Materials and Methods:**

The ovarian cortexes from 19 women with cervix and uterine malignancy aged 21–39 yr were collected. Cumulus-oocyte complexes were aspirated from all visible antral follicles. 102 immature oocytes were collected, and 43 oocytes were detected appropriately for IVM (control group). Also, 59 immature oocytes were vitrified, then matured in vitro (IVM) in two groups: with *Growth/differentiation factor 9* (GDF9) (group 1) and without GDF9 (group 2) supplementation. Rates of oocytes viability, maturation, and survival along with meiotic spindle visualization and zona pellucida birefringence were assessed with Polyscope.

**Results:**

The rate of maturation was significantly higher in controls (55.8%) compared to the other groups. Maturation rate was 23.3% in oocytes cultured in IVM medium enriched with GDF9, and 27.6% in those cultured in IVM medium lacking GDF9 (p = 0.86). Also, the meiotic spindle was present in 74.4% of control oocytes which was significantly higher than the other groups. The proportion of high zona pellucida birefringence was higher in the controls when compared with group 1 (51.2% vs. 23.3%, respectively, p = 0.04).

**Conclusion:**

Vitrification had a detrimental effect on oocyte maturation, viability as well as the subcellular quality of the oocytes after IVM in cancerous women.

## 1. Introduction

Since the diagnosis and treatment of cancerous patients have been developed successfully in recent years, fertility preservation has been specified as a priority for these patients (1). In this way, fertility preservation could be beneficial for several groups including men, women, and children with malignant diseases undergoing gonadotoxic treatment, patients affected by other non-oncologic malignancies, and age-related infertility (2). It is well-documented that chemo- and radiotherapy can result in loss of reproductive function and decline ovarian reservation in these patients (3). The methods have been already proposed to overcome the induced infertility consisting the pharmacological protection, ovarian transposition or transplantation, oophorectomy, as well as oocytes, embryos, and ovarian tissue cryopreservation (4, 5). Oocytes could be retrieved at the metaphase II (MII), metaphase I (MI), or germinal vesicle (GV) stage from the ovarian tissues (OTs). The retrieved immature oocytes may be cryopreserved in this step, and then subjected to the in vitro maturation (IVM) procedure. IVM was described as the maturation of immature oocytes in the culture media, with the specific advantages of low cost with minor side effects for the immature oocytes (6). It has been reported that vitrification of GV oocytes followed by IVM is an effective method for fertility preservation in the patients who did not involve ovulation stimulation and for cancerous cases who cannot postpone their chemotherapy program (7). Even though pregnancies and live births after human oocyte cryopreservation have been reported (8), there are still disagreements about IVM procedure with regards to the maturation stage of vitrified oocytes and its effect on oocytes fertility competence. Likewise, there are controversies with regard to the relationship between zona pellucida (ZP) birefringence as well as meiotic spindle (MS) detection and the rates of oocyte maturation and fertilization after IVM (9–11). On the other hand, *GDF9* (growth differentiation factor 9) plays a key role in the growth of granulosa cells, and oocyte differentiation. In addition, the high level of *GDF9* in human follicular fluid resulted in oocyte nuclear maturation (12).

The aim of this study was to evaluate the efficacy of vitrification on the survival, maturation, and viability rates of immature oocytes post IVM that were isolated from the cortex of OTs in cancerous patients.

## 2. Materials and Methods

In this experimental study, 32 women who were referred to the Department of Gynecology, Shahid Sadoughi Hospital, Shahid Sadoughi University of Medical Sciences, Yazd, Iran, were recruited. Our inclusion was women aged 15–45 yr with the cervix and uterine malignancy that were seeking fertility preservation. Women with bilateral ovarian cancer were excluded from the study. An ovarian biopsy or OT resection was performed during therapeutic surgery by laparotomy or laparoscopy. Due to the required urgent surgery, the day of the patients' menstrual cycle was not considered.

Thirteen women were lacking oocytes in the aspirated follicular fluid and excluded from the study. OTs were transported from the hospital to the IVF laboratory in Ham's F10+HSA medium at 4${}^{\circ }$C on ice within 15–20 min. At arrival, cumulus-oocyte complexes (COCs) were immediately aspirated from all visible antral follicles using 19-gauge syringe needles. The aspirated follicular fluid was shed directly into a Petri dish and examined for COCs under a stereomicroscope (37${}^{\circ }$C). Oocytes which were not denuded during aspiration were mechanically or chemically denuded with exposure to 80 IU/ml hyaluronidase solution (Sigma Aldrich, UK) before vitrification. It should be noted that all of the collected oocytes were immature at MI (4 oocytes) or GV (98 oocytes) stages. Oocytes were divided into three groups: Group 1; oocytes which were cultured in IVM medium supplemented GDF9 (Sigma-Aldrich, Copenhagen, Denmark) after vitrification. Group 2; oocytes which were cultured in IVM medium without GDF9 after vitrification. Group 3 (Control); fresh oocytes which were cultured in IVM medium free of GDF9.

### Vitrification

The immature oocytes were vitrified using RapidVit${}^{\mathrm{TM}}$ Oocyte kit (Vitrolife Co, Sweden) following the manufacturer's instructions; To do this, we placed 1${}^{\mathrm{cc}}$ from each of the culture mediums, “Vitri 1${}^{\mathrm{TM}}$ Oocyte and Vitri 2${}^{\mathrm{TM}}$ Oocyte”, in the wells of a multi-well plate, separately and then warmed to 37${}^{\circ }$C in environment. First, the oocytes were transferred into “Vitri 1${}^{\mathrm{TM}}$ Oocyte”. The oocytes remained in the solution for at least 5 min, and a maximum of 20 min. Then, the oocytes were moved into “Vitri 2${}^{\mathrm{TM}}$ Oocyte” for 2–5 min. The oocytes usually tended to float to the surface. If so, they were collected and placed to the bottom of the dish. Within 5 minutes, it was expected that the oocytes be re-expand to their original volume. It would indicate that the medium temperature is not at 37 ${}^{\circ }$C, if re-expansion took more than this time (5 min). After full oocyte re-expansion, we made a 20μl drop of “Vitri 3 ${}^{\mathrm{TM}}$ Oocyte” into a culture dish for an easier loading on cryodevice. After 15 seconds of oocytes transfer to the 20μl “Vitri 3 ${}^{\mathrm{TM}}$ Oocyte” droplet, they were collected and placed in the cryodevice, then immersed into liquid nitrogen.

### Warming and in vitro maturation

Warming the vitrified oocytes was performed by RapidWarm${}^{\mathrm{TM}}$ Oocyte kit, (Vitrolife Co., Sweden) following the manufacturer's instructions. For warming, the cryodevice was removed from the liquid nitrogen and dipped into Warming Solution 1 (Warm 1${}^{\mathrm{TM}}$ Oocyte), for 1 min and moved into a Warm 2${}^{\mathrm{TM}}$ for 3 min. Then, the oocytes were moved into a Warm 3${}^{\mathrm{TM}}$ for 5 min. Finally, the oocytes were transferred into Warm 4${}^{\mathrm{TM}}$ for 5–10 min. Medium volume for each step was 1 ml in 4-well dishes at 37${}^{\circ }$C. Two separate dishes containing IVM medium were prepared by a technician who was blinded to the study. GDF9 (200 ng/ml) was added to one of the IVM media (13). Sibling oocytes were transferred into two media: IVM medium with GDF9 (group 1) and IVM medium without GDF9 (group 2). The control group included the fresh immature oocytes assigned for IVM. The immature oocytes in three groups were incubated (at 37${}^{\circ }$C with 5% CO${}_{2}$ and 95% air, with high humidity) in IVM Medium (MediCult IVMⓇ System, Pr, Origio company, Denmark) supplemented with 75 mIU/ml FSH, 100 mIU/ml hCG, and 20% human serum albumin (HSA) added to vial two of the media. The oocytes were then evaluated for maturity between 24 and 48 h after IVM. Oocyte maturation was assessed by the presence of the first polar body under a stereomicroscope (Nikon, Japan). Oocytes were considered morphologically survived after warming by the presence of a round shape and size along with the intact ZP, and clear perivitelline space, with no sign of degeneration.

### Meiotic spindle and ZP birefringence evaluation

**Figure 1:**
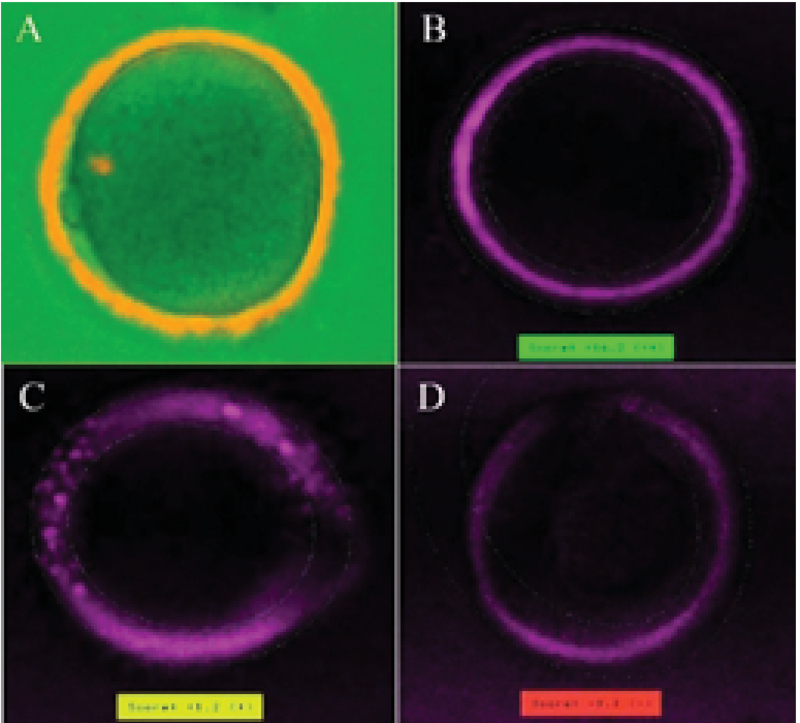
Meiotic spindle (MS) and ZP birefringence evaluation. A: Meiotic spindle in the mature oocyte. B, C, and D: ZP birefringence (B: Green; C: Yellow; D: Red) (40× magnification).

Meiotic spindle and ZP birefringence were examined using PolScope as described previously (14). After placing the mature oocytes, individually, in an well-equilibrated 3-µl droplet of buffered medium (G-Mops-V1; Vitrolife), in a glass-bottomed culture dish (WillCo-Dish; Bellco Glass, USA), the presence of MS and homogeny of the inner layer of the ZP were evaluated using a polarizing optical system (OCTAX PolarAIDE; Octax, Germany). According to the results determined by the software of the PolarAid system, the oocytes were stratified into two classifications of presence or absence of MS. Also, three categories of high ZP (green), moderate ZP (yellow) and low ZP birefringence (red) were assigned (15) (Figure 1).

### Assessment of viability

**Figure 2:**
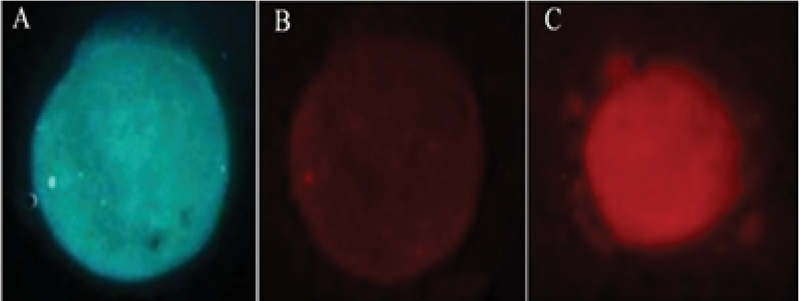
Viable staining of the oocytes. A: live oocyte with intact cell membrane was shown blue fluorescence (PI-negative). B: live oocyte with intact cell membrane. C: Dead cell (PI-positive) (20× magnification).

Oocytes viability assessment was performed with Hoechst/propidium iodide (H/PI) nuclear staining based on a previous report (16). Briefly, the oocytes were washed twice in prewarmed Ca${}^{+2}$ and Mg ${}^{+2}$ free phosphate buffer saline (PBS-). Then, they were incubated in 20 µg/ml of H33342 (Sigma, USA) and 20 µg/ml of PI (Sigma, USA) for 15 min. After that, the oocytes were washed thrice in warm PBS until the residual dye was resolved. The cells were examined under a fluorescence microscope (Olympus; Japan). The fluorescent dye enters both alive and dead cells, while PI just enters the cells with altered membrane integrity. Therefore, alive cells with intact cell membrane showed blue fluorescence (PI-negative) and cells with late apoptosis and necrosis appeared as red (PI-positive) (Figure 2).

### Outcome measurements

The primary outcome was the oocyte maturation rate. Oocyte maturation was assessed by the presence of the first polar body under a dissecting microscope. Oocyte survival rate and viability were assessed as secondary outcomes. Survival of the oocyte was defined as regular oocyte shape and diameter, observing intact ZP and oolemma, a clear perivitelline space and no sign of ooplasmic degeneration. For oocyte viability, the dead and the viable cells were identified according to the PI status. The correlation between women age and the number of recovered oocytes was calculated.

### Ethical consideration

The patients voluntarily participated in the study and signed the written informed consent before the procedure. The study was reviewed and approved by the Ethics Committee of Shahid Sadoughi University of Medical Sciences, Yazd, Iran (IR.SSU.RSI.REC.1395.1).

### Statistical analysis

Statistical analysis was performed using the Statistical Package for the Social Science version 20.0 (SPSS Inc, Chicago. IL, USA). The data were analyzed using the Chi-square test. A Pearson rank correlation was used to analyze the relationships between age and the number of oocytes. P-value of < 0.05 was considered significant.

## 3. Results

Totally, 102 immature oocytes were obtained with the mean of 5.37 ± 3.14 oocytes per patient. The mean age of women was 31.74 ± 5.06. The women age negatively impacted the number of oocytes obtained from the ovarian samples. However, there was no significant statistical correlation between the age and the number of recovered oocytes (rs = –0.34; p = 0.15). The percentage of mature oocytes was significantly higher in the control group (55.8%) compared to the other groups undergoing IVM after vitrification. Maturation rate was 23.3% in oocytes cultured in IVM medium enriched with GDF9 and 27.6% in those cultured in IVM medium lacking GDF9 (p = 0.86). Also, the rates of arrested and degenerated oocytes were lower in controls than other groups (Table I). The data showed that survival rate in addition to the viability of oocytes after vitrification was comparable between groups 1 and 2 (Table I). The findings indicated that MS visualization was present in 74.4% of the oocytes in controls which was significantly higher than the other groups. Nevertheless, the percentage of visualized MS was similar between groups 1 and 2 (Table II). The proportion of high ZP birefringence was higher in the control group compared with the IVM group plus GDF9 (51.2% vs. 23.3% respectively, p = 0.04). In addition, the lower rate of moderate ZP birefringence attained in the control group (25.4%). But the difference did not reach a significant level in comparison with the other groups. Low ZP birefringence was comparable among the three groups (Table II).

**Table 1:** Maturation rate, survival rate, and viability of oocytes in three groups


**Variable**		**Group 1 (With GDF9) (n = 30)**	**Group 2 (Without GDF9) (n = 29)**	**Group 3 (Control Group) (n = 43)**	**P-value**
Maturation rate					
	Mature	7 (23.3)	8 (27.6)	24 (55.8)	0.02${}^{a}$
	Arrest	14 (46.7)	14 (48.3)	12 (27.9)	0.04${}^{b}$
	Degeneration	9 (30)	7 (24.1)	7 (16.3)	0.86${}^{c}$
	Survival rate	21 (70)	20 (69)	–	0.93${}^{c}$
Viability					
	Alive	22 (73.3)	21 (72.4)	–	0.93${}^{c}$
	Dead	8 (26.7)	8 (27.6)	
Note: Data are presented as number (%); Differences between groups using Chi-squared test; a: group 1 vs. group 3; b: group 2 vs. group 3; c: group 1 vs. group 2; GDF9: Growth differentiation factor-9					

**Table 2:** Meiotic spindle visualization and Zona pellucida birefringence among three groups


**Variable**		**Group 1 (With GDF9) (n = 30)**	**Group 2 (Without GDF9) (n = 29)**	**Group 3 (Control Group) (n = 43)**	**P-value**
Meiotic spindle					0.03${}^{a}$
	Visible	2 (6.7)	2 (6.9)	11 (25.5)	0.04${}^{b}$
	Invisible	28 (93.3)	27 (93.1)	32 (74.4)	0.97${}^{c}$
Zona pellucida					
	Green	7 (23.3)	7 (24.1)	22 (51.2)	0.04${}^{a}$
	Yellow	14 (46.7)	14 (48.3)	11 (25.6)	0.05${}^{b}$
	Red	9 (30)	8 (27.6)	10 (23.3)	0.97${}^{c}$
Note: Data are presented as number (%); Differences between groups using Chi-squared test; a: group 1 vs. group 3; b: group 2 vs. group 3; c: group 1 vs. group 2; GDF9: Growth differentiation factor-9.					

## 4. Discussion

In spite of significant improvements in IVM technique and progress in pregnancy and implantation rates with in vitro matured oocytes, the need for more objective and accurate investigation has been encouraged. For the first time, we evaluated the post-thawing outcomes of vitrified immature oocytes collected from the excised ovarian tissue. Our study showed that vitrification of immature oocytes before IVM has a detrimental effect on maturation rate during IVM program. We found no significant correlation between age and the number of recovered oocytes. Similarly, another study reported no significant correlation between the number of the collected oocytes from the ovarian medulla tissue and the patients' age (17). Whereas Fasano and colleagues showed a significant negative correlation between the number of retrieved oocytes and female age. They collected a significantly higher number of oocytes from the OT of the pre-pubertal girls compared with adults (18). Other studies implemented in infertile patients about the age of 30 yr have stated that 6–20 retrieved oocytes during ovarian stimulation may improve the live-birth rate (19, 20). However, these results are reported from controlled ovarian stimulation cycle, and could not generalize to IVM setting.

As the main outcome, the maturation rate was significantly higher in the oocytes that did not vitrified compared with vitrified oocytes. Several reports indicated that oocyte maturation rate was significantly higher for oocytes matured before vitrification than those vitrified before IVM (6, 16). In addition, our recent review and meta-analysis revealed that oocyte vitrification decreases the maturation rate significantly but survival and fertilization rates along with cleavage rat did not significantly vary between the oocytes vitrified before and after IVM (21). In addition, we found similar results in our previous report; also, the maturation rate in women under the age of 30 was higher than older patients in both fresh and vitrified IVM (22). In this way, Yin and colleagues vitrified MII oocytes after IVM and found that maturation rate in patients below 20 years was significantly higher than patients aged under or above 30 yr (17). In general, previous studies demonstrated that cryopreservation procedure has a negative effect on oocyte maturation capacity (23), particularly when the oocytes were vitrified in immature GV stage and then subjected to IVM (24). It is documented that some disorders may occur during the vitrification process in different cellular components, such as cytoskeleton abnormalities as well as chromosome separation instabilities and calcium signaling system disturbances (25). It is also shown that vitrified MI oocytes are more capable to reach MII stage after warming when compared to vitrified GV oocytes. It may be due to the first step of maturation which is more vital and mainly affected by vitrification (26). A good option in this subject may define as embryo formation after IVM followed by embryo vitrification. Our previous study showed that the immature oocytes retrieved from the ovarian tissue of cancer women could successfully subject to IVM for the generation of good quality and freezable embryos using intracytoplasmic sperm injection (ICSI) (27).

Moreover, we evaluated the effect of GDF9 supplementation on oocyte maturation. Interestingly, we observed that oocyte maturation rate was comparable between two groups of frozen-thawed oocytes which treated or not treat with GDF9. But, significantly higher maturation rate was detected in the control group compared to the two aforementioned groups. It is well documented that GDF9 is involved in the folliculogenesis, also in the final stages of maturation, as mutations or deletion of GDF9 gene is associated with fertility problem and reproductive defects in rats, mice, sheep, and humans (28). Our results may demonstrate that vitrification play a more important role in oocyte maturation in comparison with GDF9 supplementation. Conversely, other studies reported the improvement of ovarian follicle growth and differentiation (29) along with subsequent embryo development in mice (13). Likewise, the relationship between the levels of GDF9 and nuclear oocyte maturation has been shown in human (12). Similarly, Hreinsson and colleagues showed that GDF9 enhanced the growth, development, and survival of human ovarian follicles (30).

In addition, we evaluated the oocyte survival rate after vitrification which was similar to group 1 (70%) and 2 (69%). The results showed that the vitrification procedure had no negative effect on the survival rate regardless of GDF9 supplementation. Likewise, two other studies reported oocyte survival rate of 64% (17) and 61.5% (31) after vitrification of GV and MII oocytes, respectively. It is also indicated previously that survival rate after warming is comparable between oocytes that vitrified in GV and MII stages (24). Similarly, animal studies reported high survival rates after oocyte vitrification and concluded that oocyte vitrification is a very effective method for cryopreservation of mouse and rat oocytes (32, 33). It is well known that during the vitrification process, the oocytes are exposed to high cooling rates along with high cryoprotectant concentrations for preventing ice-crystal formation and increasing the oocyte survival rate (34, 35). We also assessed the viability of oocytes in the presence or absence of GDF9. The results showed the similar viability rates between the oocytes in two groups. Recently, Yazdanpanah and co-workers compared post-IVM viability rate between 53 immature oocytes in fresh and 50 immature oocytes in the vitrification group. They stated a significantly reduced viability rate in vitrified IVM group (16).

In the present study, ZP birefringence and MS visualization were assessed as oocyte quality markers after vitrification and IVM. The percentages of high ZP birefringence and visualized MS were significantly higher in the oocytes without vitrification in comparison with vitrified oocytes with or without GDF9 treatment. It has been well documented that ZP birefringence and MS visualization could be considered for evaluation of oocyte developmental capacity and subsequent ART outcome (10, 11, 36). Lierman and colleagues compared spindle presence in the vitrified and non-vitrified IVM oocytes collected from the medulla suspension of trans-men. They reported a non-significant presence of bipolar spindles in both vitrified and non-vitrified group by over than 50% (37). Two other studies evaluated ZP and MS among in vivo and in vitro matured oocytes and stated the higher percentages of high ZP birefringence in IVM oocytes in addition to similar MS visualization between groups (10, 11). Previous studies indicated that IVM has not negatively affected ZP birefringence (38, 39); however, our results showed that vitrification may have a detrimental effect on oocyte quality through alteration in ZP and MS condition.

## 5. Conclusion

In conclusion, vitrification had the most detrimental effect on oocyte maturation and viability as well as ZP birefringence and the presence of MS in the oocytes. Therefore, vitrification of mature oocytes after IVM may be more promising. Optimization of the vitrification procedure in addition to embryo cryopreservation is the most reputable method for cancerous patients.

##  Conflict of Interest 

There is no conflict of interest regarding our results.
